# Incidence of aflatoxin M_1_ in cows’ milk in Pakistan, effects on milk quality and evaluation of therapeutic management in dairy animals

**DOI:** 10.17221/18/2023-VETMED

**Published:** 2023-06-21

**Authors:** Inayat Ullah, Amar Nasir, Muhammad Kashif, Arbab Sikandar, Muhammad Sajid, Muhammad Adil, Aziz ur Rehman, Muhammad Umer Iqbal, Habib Ullah

**Affiliations:** ^1^Department of Clinical Sciences, Sub-Campus Jhang, University of Veterinary and Animal Sciences (UVAS), Lahore, Pakistan; ^2^Department of Basic Sciences, Sub-Campus Jhang UVAS, Lahore, Pakistan; ^3^Department of Pathobiology, Sub-Campus Jhang UVAS, Lahore, Pakistan; ^4^Livestock and Dairy Development Department, Government of Punjab, Pakistan; ^5^Faculty of Veterinary and Animal Sciences, Gomal University, D. I. Khan, Pakistan

**Keywords:** dairy animals, mycotoxin, toxin binder, wholesomeness of milk season

## Abstract

The present study was aimed at measuring the concentration of aflatoxin M_1_ (AFM_1_) in the milk of Holstein Friesian cows, its effect on the milk quality and seasonal trends, as well as to investigate the efficacy of a commercial clay-based toxin binder. For this purpose, milk samples from dairy cows (*n* = 72) were collected and assayed for AFM_1_ before employing a clay-based toxin binder. The milk samples (*n* = 72) were collected from selected animals, revealing that 69.4% of the milk samples had AFM_1_ levels above the United States permissible limit (0.5 μg/kg). The incidence of AFM_1_ in milk during the winter and summer was 82.5% and 53.1%, respectively. Owing to the presence of AFM_1_, the level of milk fat, solids-not-fat, and protein were found to be low. Subsequently, the affected animals were divided into two groups, i.e., AFM_1_ positive control (*n* = 10) and the experimental group (*n* = 40). The experimental group of animals were fed the clay-based toxin binder at 25 g/animal/day. A progressive decrease of 19.8% in the AFM_1_ levels was observed on day 4 and on day 7 (53.6%) in the treatment group. Furthermore, the fat, solids-non-fat and protein increased significantly in the milk. In conclusion, a high level of AFM_1_ contamination occurs in the milk in Pakistan, affecting the quality of the milk production. Clay-based toxin binders may be used to ensure the milk quality and to protect the animal and consumer health.

Aflatoxins are a group of mycotoxins, produced as toxic secondary metabolites of fungi, which cause a range of harmful effects in vertebrates. They are formed in feedstuff-like roughage and concentrates due to improper storage. Aflatoxin B_1_ (AFB_1_) accumulates in the rumen of the animals that consume contaminated feed (Esam et al. 2022; Odjo et al. 2022) and is later absorbed through the digestive tract. AFB_1_ is transformed into AFM_1_ in hepatocytes by microsomal cytochrome P450 and is subsequently eliminated via the milk (Battacone et al. 2005). In addition, AFB_1_ reactive peroxides are also formed during this reaction. Milk components of dairy animals, particularly fat, solids-not-fat and proteins, are affected by the presence of AFM_1_ and their levels are reduced (Yousof and El Zubeir 2020). This reduction in the milk quality has also been linked to the compromised immune response in dairy cows due to the adverse effect of AFM_1_ (Garvican et al. 1973; Barbiroli et al. 2007; Queiroz et al. 2012).

Feed contamination with aflatoxin varies seasonally, such as that during the summer when the temperature is high, and in autumn when the environment is humid, the conditions are more favourable for the production of mycotoxins in the feed (Tavakoli et al. 2013). Milk is an essential component of the food pyramid and a rich source of calcium and protein. AFM_1_ is highly detrimental to human health as well as animal health, though less toxic than aflatoxin B_1_ (Esam et al. 2022; Wang et al. 2022). AFM_1_ may cause both acute and chronic toxicosis and there is worldwide concern about its liver and kidney associated carcinogenic properties and other health-related issues for milk consumers (Tajkarimi et al. 2008; Li et al. 2018). A risk of stunting is also possible through AFM_1_ consumption. The impact of aflatoxin M_1_ on the age, gender, season, and nutritional behaviour of the patient varies. Low production, sub-clinical mastitis, low rumen motility, blood coagulation defects, hepatotoxicity, immune suppression, and mutation are the significant effects of aflatoxins on an animal’s health. It is also pertinent that breast feeding has also been linked with the presence of aflatoxin M_1_ in infants (Hussen Kabthymer et al. 2023).

Milk and milk products have a limit of up to 0.5 μg/kg of AFM_1_ only in order to be acceptable for consumption in Pakistan (Aslam et al. 2015). Contamination of milk with AFM_1_ may be addressed either directly, by decreasing the AFM_1_ content in the contaminated milk, or indirectly, by decreasing the AFB_1_ contamination in the feed of the dairy animals.

Toxin binders have been used to reduce the presence and effect of AFM_1_ (Kuboka et al. 2022). Hydrated sodium calcium alumino-silicates (HSCAS), bentonites, zeolite, and charcoal are good clay-based toxin binders (Farkas et al. 2022). In the animal body, toxin binders work in two ways: The first one is through the complex formation process in the gastrointestinal tract of the animal. Then, these complexes are excreted through the faeces resulting in the reduction of the bioavailability of toxins (Mutua et al. 2019). On the other hand, an alternative method is to capture aflatoxin B_1_ and alter its chemical structure. In this way, aflatoxin B_1_ cannot be changed into AFM_1_ in the milk. Some activated carbons are frequently used because they bind and eliminate the effects of the mycotoxins (Lo Dico et al. 2022). While the efficacy of these activated carbon compounds is inconsistent; bentonites are well-sought-out as an adsorbent for mycotoxins (Vekiru et al. 2007).

The presence of aflatoxins in milk impacting the quality of dairy products is the most important present problem in the Pakistan dairy industry. Local dairy farmers are experiencing economic losses due to the low milk prices being paid on account of the aflatoxin-affected milk. The present study was, thus, designed to evaluate the level and seasonal trends of aflatoxin M_1_ in milk, its effect on the milk quality and to evaluate a means of mitigating the mycotoxins through a specific clay binder.

## MATERIAL AND METHODS

### Experimental design

Milk samples from 72 lactating Holstein Friesian cows (in early to mid-lactation ranging from 2^nd^ to 5^th^ parity) were randomly obtained from ten selected dairy farms, during the months from December, 2018 to June, 2019) in the districts Jhang and Layyah, Punjab, Pakistan. A total of 40 milk samples were collected during the winter months (December to February), while 32 samples were procured during the summer (March to June). All the studied animals were reared under semi-controlled farming systems. The number of animal samples obtained per farm ranged from 3–6. Animals with AFM_1_ levels above the threshold value were included in the therapeutic trial.

The laboratory of Haleeb Foods Pvt. Ltd. located in Layyah served as the facility for the analysis to detect AFM_1_ in the milk. Out of the 72 milk samples, 50 milk samples (*n* = 50) had AFM_1_ above the permissible level limit (0.5 μg/kg), according to the United States (US) standard, thus a therapeutic trial was performed on those animals (*n* = 50). Ten animals were kept as a clinically positive control, the rest of the animals (*n* = 40) were reserved in the experimental group and were fed the mycotoxin binder Mastersorb Premium^®^ (EW Nutrition, Germany) at a rate of 25 g/day per animal during the trial period, administered in a bolus form following the dose recommendation of the product manufacturer. All the animals (*n* = 50) (both the control and experimental ones) in the current study were fed on the same feeding plan.

### Sampling protocols

During the morning and/or evening milking, 250 ml of fresh milk samples were collected from each of the 72 lactating cows in single-use disposable plastic vials (ALWSCI Technologies, Shanghai, P.R. China) with tight-fitting screw caps for screening the level of AFM_1_ in the animals. Subsequently, the milk samples of the selected cows were collected on day 0, day 4, and day 7 post-feeding the mycotoxin binder Mastersorb Premium^®^. The samples were properly labelled and transported in a cold chain to the laboratory and were screened on the same day.

### Sample processing

The manufacturer’s instructions were followed in processing the samples. Milk samples were poured into microtubes for centrifugation at 136 relative centrifugal force (rcf) for 5 min at room temperature (27 °C). Three hundred microlitres (300 μl) of the milk sample was taken and, after the fat was removed in a micro tube, the samples were refrigerated. An SL Aflatoxin M1 Quantitative Test dilution buffer was added to the micro tube containing the milk sample. Afterwards, it was plugged into a Charm EZ Lite unit (https://mcsdiagnostics.com/product/charm-ez-lite-system/) waiting for the “Insert Strip to Start” screen. The test strip was placed in the Charm EZ Lite unit. This system works on the principle of immuanuanosensors comprised of bioreceptors, a transducer and an electronic system. The bioreceptor may act as an antibody or enzyme combining with the analyte/aflatoxin present in the milk sample and this interaction leads to a change in the photon, electron, masses, heat or pH. This change is transformed into measurable waves by a transducer and is interpreted by the electronic system (Wang et al. 2016; Majdinasab et al. 2020). The Charm EZ Lite unit automatically reads the test strip and adjusts the assay type and incubator temperature to match the inserted test. The limit of detection and limit of quantification of the instrument are 0.2 μg/kg and 0.75 μg/kg, respectively. Finally, the reading on the screen showed the level of AFM_1_ that was recorded. Each of the collected samples were tested twice and their obtained mean value was used for further analysis. The Charm EZ Lite system instantly identifies the AFM_1_ residue by the test colour on the rapid one step assay strips. The Charm EZ Lite system automatically adjusts the temperature, test calibration and incubation time. It has been designed to simplify the testing, reduce any operator error and provide real time results of AFM_1_ (Urusov et al. 2019).

### Milk quality indicators

To evaluate milk quality, the proportion of fat, solids-non-fat (SNF) and protein were checked. The fat content was measured by the Gerber method. The total solids (TS) and SNF were estimated by using Richmond’s formula, as follows (Lopez et al. 1991):

TS (%)=CLR/4+1.21 F+0.14 (1)

SNF (%)=CLR/4+0.21 F+0.14 (2)

where:

CLR – the corrected lactometer reading;

F – the fat content of the milk.

The milk protein was evaluated by using a phenolphthalein indicator (protein % = V1 – V2 × 1.94) – by this formula, the normal value for the milk protein, being 0.32–0.37 V1, is the initial reading of the burette and V2 is the final reading where the colour changes during titration with N/10 NaOH (James 2013).

### Statistical analysis

The obtained data regarding the different parameters of the study (incidence of AFM_1_, effect of the mycotoxin binder and quality of milk before and after applying the mycotoxin binder) from the current study was normally distributed in the sample size. To detect the incidenceof AFM_1,_ the Pearson Chi-square test was used. Repeated measurements of the milk samples to detect the concentration of AFM_1_ on days 0, 4 and 7 were conducted using a repeated measures analysis of variance (ANOVA). The effect of AFM_1_ on the milk quality parameters (fat, SNF and protein) was calculated using the paired *t*-test using SPSS software v21.0 (IBM, USA). The level of significance for the statistical analysis was determined at a 5% probability level (*P* < 0.05).

## RESULTS

### District-wise prevalence of AFM_1_ in dairy cows

Out of the tested 72 milk samples, 75.6% (28/37) of the milk samples had AFM_1_ levels above the permissible AFM_1_ limit (0.5 μg/kg) in the Jhang district, while 62.8% (22/35) of the milk samples had AFM_1_ levels above the permissible limit in the Layyah district. The overall incidence of AFM_1_ above the permissible limit in all 72 samples was 69.4% (50/72) (Table 1).

**Table 1:** Prevalence of AFM_1_ in the milk samples of 72 dairy cows in the Jhang and Layyah districts

District	No. of dairy cows tested (*n*)	No. of positive animals (> 0.5 μg/kg)	Prevalence % (at 95% CI)	*P*-value
Jhang	37	28	75.6 ± 13.8	0.238
Layyah	35	22	62.8 ± 16.0	
Total	72	50	69.4 ± 10.6	

CI = confidence interval

### Seasonal impact of AFM_1_

In the winter, 82.5% of the milk samples (33/40) had AFM_1_ levels above the limits. In the summer months, 53.1% of the milk samples (17/32) had AFM_1_ levels above the recommended safe limits in the two districts of the study (Table 2).

**Table 2:** Seasonal impact of AFM_1_ in the dairy cows in the Jhang and Layyah districts

Season	No. of dairy cows tested (*n*)	No. of positive animals (> 0.5 μg/kg)	Prevalence % (at 95% CI)	*P*-value
Winter	40	33	82.5^a^ ± 11.8	0.007
Summer	32	17	53.1^b^ ± 17.1	
Total	72	50	69.4 ± 10.6	

^a–b^Means within the same column marked with different superscripts were significantly different (*P* < 0.05) using the Pearson Chi square test

CI = confidence interval

### AFM_1_ reduction on the different days of the study

After the milk analysis, for the AFM_1_ affected samples, a clay-based toxin binder (Mastersorb^®^) was used as a therapeutic agent and its efficacy was measured.

During the trial, a reduction of 19.8% in the level of AFM_1_ was recorded on day 4 and a 53.6% reduction was recorded on day 7 post-administration (Table 3).

**Table 3:** Reduction (%) of AFM_1_ among the dairy cows of the two districts after using the toxin binder (Mastersorb^®^) over a period of 7 days

Status of aflatoxin M_1_ in animals	Day 0 mean ± SD (μg/kg)	Day 4 mean ± SD (μg/kg)	Day 7 mean ± SD (μg/kg)	*P*-value
Clinically affected experimental animals (*n* = 40)	0.741 ± 0.12	0.594 ± 0.08	0.344 ± 0.03	0.000
Reduction (%)	0^a^	19.8^b^	53.6^c^	
Clinically affected control animals (*n* = 10)	0.617 ± 0.03	0.616 ± 0.03	0.614 ± 0.03	0.086
Reduction (%)	0	0.17	0.49	

^a–c^Means ± SD within a row marked with different superscripts were significantly different (*P* < 0.05) using a repeated measures ANOVA

SD = standard deviation; μg/kg = microgram per kilogram

### Effects of AFM_1_ on the milk fat, SNF and protein

The presence of aflatoxin M_1_ is the sole agent which affected the quality of the milk by lowering the fat, SNF and protein levels (Table 4).

**Table 4:** Milk fat, solids-not-fat and protein concentration before and after the toxin binder administration (Mastersorb^®^) in forty animals from dairy farms in the Jhang and Layyah districts

Dairy farm location	Milk quality parameters	Before treatment (mean ± SD)	4^th^ day post-administration (mean ± SD)	*P*-value
Animals in district Jhang	Fat (%)	3.82 ± 0.06^a^	4.09 ± 0.05^b^	0.001
	Solids-not-fat (SNF) (g/l)	8.09 ± 0.26^a^	8.28 ± 0.18^b^	0.016
	Milk protein (g/l)	32.79 ± 1.24^a^	32.90 ± 1.30^b^	0.050
				
Animals in district Layyah	Fat (%)	3.84 ± 0.07^a^	4.11 ± 0.06^b^	0.000
	Solids-not-fat (SNF) (g/l)	7.95 ± 0.26^b^	8.20 ± 0.32^a^	0.000
	Milk protein (g/l)	33.02 ± 1.22^a^	33.12 ± 1.28^b^	0.040

^a–b^Means ± SD within the same row marked with different superscripts were significantly different (*P* < 0.05)

SD = standard deviation

### Use of mycotoxin binder (Mastersorb^®^) in dairy cows

In the present study, the Mastersorb^®^ toxin binder displayed a significant impact in neutralising the effect of aflatoxin M_1_ in the milk and improved the quality of the milk (Table 5).

**Table 5:** Aflatoxin M_1_ level before and after using the toxin binder in the district-based dairy farms of Jhang and Layyah

Number of animals tested	Aflatoxin M_1_ level (μg/kg)		
	Before treatment (day 0) (mean ± SD)	Post-administration (day 4) (mean ± SD)	*P*-value
Jhang (*n* = 23)	0.72 ± 0.05^a^	0.46 ± 0.14^b^	0.000
Layyah (*n* = 17)	0.77 ± 0.04^a^	0.47 ± 0.16^b^	0.002

^a–b^Means ± SD within the same row marked with different superscripts were significantly different (*P* < 0.05) using the paired samples *t*-test

SD = standard deviation; μg/kg = microgram per kilogram

## DISCUSSION

In dairy milk, AFM_1_ is one of the most important carcinogenic agents if consumed in high levels, especially for the old and young (Tadesse et al. 2020). The International Agency for Research on Cancer has graded both AFB_1_ and AFM_1_ as Group-1 carcinogens (IARC 2002). Our study has shown that 69.4% of the cow milk samples had AFM_1_ quantities higher than the US AFM_1_ defined limit (for consumer health) of 0.5 μg/kg, using the Charm EZ Lite^®^ process, a lateral flow immune-sensor method, rapid one-step assay. Another study conducted by Hussain and Anwar (2008) to determine the AFM_1_ levels using high performance liquid chromatography (HPLC) showed that 42.5% of buffalo and 52.5% of cow milk were contaminated (levels above the threshold) with AFM_1_. In the summer season, the levels of aflatoxin M_1_ were recorded as being lower (53.1%) than the winter season (82.5%) in the present study. The main reason behind this is the elevated moisture level in the winter season. It has been commonly observed that humidity makes the environment conducive for the growth of moulds and subsequently deteriorates the quality of the feedstuff stored in such conditions. A similar experiment was conducted elsewhere in which 360 milk samples were collected from different species (buffaloes, cows, sheep and goats) over a period of 12 months (Ismail et al. 2016). The results of this study are in general agreement with the findings of our study, showing 56% of cow milk samples contaminated with AFM_1_ in the winter months, while only 38% were contaminated in the summer. A similar trend in the seasonal incidence of AFM_1_ in dairy milk samples has also been reported in another study carried out in 14 states in Iran, with a significantly higher aflatoxin level in the summer than the winter (Tajkarimi et al. 2008).

The environmental conditions of the Punjab area of Pakistan are conducive for fungal growth (Asi et al. 2012). Most studies have demonstrated high AFM_1_ concentrations in agreement with our findings. This is most likely because, in Pakistan, the feed is stored in compounds with high moisture contents and, hence, a high possibility of the presence of AFB_1_ is then offered to dairy animals. In contrast to our results, a higher concentration of AFM_1_ was reported in the autumn and monsoon season by Aslam et al. (2016). The differences in the seasonal prevalence may be due to the geo-climatic variations in the study areas as the referred study was carried out in the districts of the upper Punjab area including Kasur, Lahore, Pakpattan and Okara with higher rainfall during the mentioned seasons than in the Southern Punjab areas of the current study (Jhang and Layyah Districts).

We used a clay-based toxin binder (Mastersorb^®^) and found progressive decreasing levels of AFM_1_ to day 7, ultimately reducing the AFM_1_ levels below the threshold for all the cattle. Thus, it is evident that the use of the toxin binder Mastersorb^®^ proved helpful in controlling AFM_1_ in the milk (Table 3). Aflatoxin can bind to some fractions of milk protein (Barbiroli et al. 2007) causing a reduction in the milk protein level, which may possibly be associated with interference in the synthesis of milk components (Garvican et al. 1973). The ingested AFM_1_ may also deteriorate the innate immunity leading to a decline in the milk ingredients (Queiroz et al. 2012; Yousof and El Zubeir 2020). The same has been reported by Nasir et al. (2022) and Faraz et al. (2013) after obtaining milk samples from the industrial areas of Faisalabad, Division, Pakistan. They reported lower levels of protein, fat and SNF in the milk samples besides detecting milk adulterants (formalin, cane sugar, hydrogen peroxide and urea), as well as AFM_1_.

A number of previously published reports are available in the literature (Hussain and Anwar 2008; Asi et al. 2012; Faraz et al. 2013; Aslam et al. 2016) relating to the AFM_1_ prevalence in dairy products in Pakistan, but no one has specifically highlighted the use of a clay-based toxin binder in the animal diet. Some reports in the literature are available regarding effective biological, chemical, or physical means of degradation of the mycotoxins in the feed (Peng et al. 2018). A product called Mycofix^®^ Plus (DSM Animal Health and Nutrition, Austria) is also a very popular product being used in animal diets to counter the deleterious effects of mycotoxins (Kiyothong et al. 2012; Nabi et al. 2018). Extracts of *Ascophyllum nodosum* (sea algae) and *Silybum marianum* (plant) work to counter the unfavourable environment produced by the mycotoxins (Pietri et al. 2009).

Due to the use of the toxin-binding clay, the fat, solids-not-fat and protein values increased significantly (*P* < 0.05) in the milk. Due to the functional properties and nutritional value of the mentioned milk parameters (fat, solids-not-fat and protein), milk is regarded as the most important component in a human’s diet (Hussen Kabthymer et al. 2023). The presence of aflatoxin M_1_ in the milk leads to a decrease in the SNF level causing the loss of milk texture due to lower solid concentrations (ash and minerals), hence, the nutritive value of milk becomes compromised due to the deleterious effect. After using Mastersorb^®^, an improvement was seen in the overall milk quality. Queiroz et al. (2012) also reported decreased milk protein and fat concentrations in the AFM_1_ of Holstein dairy cows in Gainesville, USA during an experimental trial. They compared the control diet with the clay-based toxin binder diet (Calibrin A; Amlan International, Chicago, IL, USA), and found significantly (*P* < 0.05) improved levels of milk fat and proteins in the treated cows. Kiyothong et al. (2012) stated that the Mycotoxin deactivator product supplemented diet significantly improved the feed intake, milk production and milk proteins in mycotoxin affected cows.

In conclusion, the bovine milk produced in the districts of Jhang and Layyah is largely contaminated with AMF_1_. Dairy cows are at a greater risk of AFM_1_ contamination during the winter. Daily feeding of a clay-based mycotoxin-binder (Mastersorb^®^) can effectively overcome this issue, resulting in the production of safe, wholesome milk. Educating dairy farmers about good feeding practices and the value of the addition of clay-based additives through extension activities will be worthwhile.
